# Part of the research family

**DOI:** 10.7554/eLife.36707

**Published:** 2018-04-04

**Authors:** Emma Pewsey

**Affiliations:** FeatureseLifeCambridgeUnited Kingdom

**Keywords:** scientist and parent, women in science, diversity, careers in science

## Abstract

eLife is publishing a collection of articles and interviews with scientists who are parents to explore how to get the best of both worlds.

Children and research projects have a lot in common. Both can lead to sleepless nights, both require the mastery of specialist techniques, and people who are not parents or scientists often struggle to understand them. However, despite these similarities, family life and a career in research are often portrayed as being incompatible with each other.

To shed light on this topic we have interviewed over 20 scientists who are also parents: these interviews will appear on the eLife website over the next few weeks, alongside articles that take a broader look at the highs and lows of being a scientist and a parent. It is striking how different each parent’s experience is, even among researchers at similar locations and career stages. Yet, as summarized here, a number of common themes emerged in the interviews, notably with regard to childcare, time, travel and flexibility.

## Where’s the childcare?

Some of the problems faced by scientists who are parents will be familiar to parents of all professions. A key pressure point is the cost and availability of childcare, and many researchers would like on-campus childcare to be provided. However, even researchers fortunate enough to work for an institute that provides childcare facilities face barriers to using them. On-campus nurseries often have months-long waiting lists, making early registration essential. “I know of a couple who booked their child in before the baby was even conceived”, says Marta Garrido of the University of Queensland. Marta herself had to wait two years for a full-time place for her first son, having registered him six months before he was born.

The costs of the facilities can also be prohibitive, particularly for early-career researchers. And even with subsidies, the cost of full-time care for one child often represents over half of a researcher’s salary. That’s when they’re able to use the facilities at all: postdoctoral researchers paid through scholarships may not be officially employed by the institution they work at, and so are ineligible for on-site nurseries provided for the children of staff and students. This situation is one example of the wider disparities in the benefits on offer to different classes of postdoctoral researchers, which have led to calls for the employment contracts of postdocs to be made more consistent (Schaller et al., 2017).

## Time out

The parental leave on offer to researchers varies widely. National standards and the gender of the parent are the main factor controlling the length of leave available, although the situation is more complicated for graduate students and postdocs on fixed-term contracts. Furthermore, parents on short-term contracts that come to an end around the time of birth can find themselves unemployed instead of on leave. Even without these pressures, researchers may choose to work through their leave period: papers and funding applications need to be written, and junior lab members need mentoring. Although the collaborative nature of science means that others may be able to help with some tasks, it can also make parents feel like they’re taking advantage of their colleagues if they take leave.

A number of initiatives offered by employers, funders and other organizations aim to provide parents with more time for research once they return from maternity leave. For example, Gaby Da Silva Xavier of Imperial College used an Elsie Widdowson Fellowship from the university to employ a technician to help run her lab.

**Figure 1:**
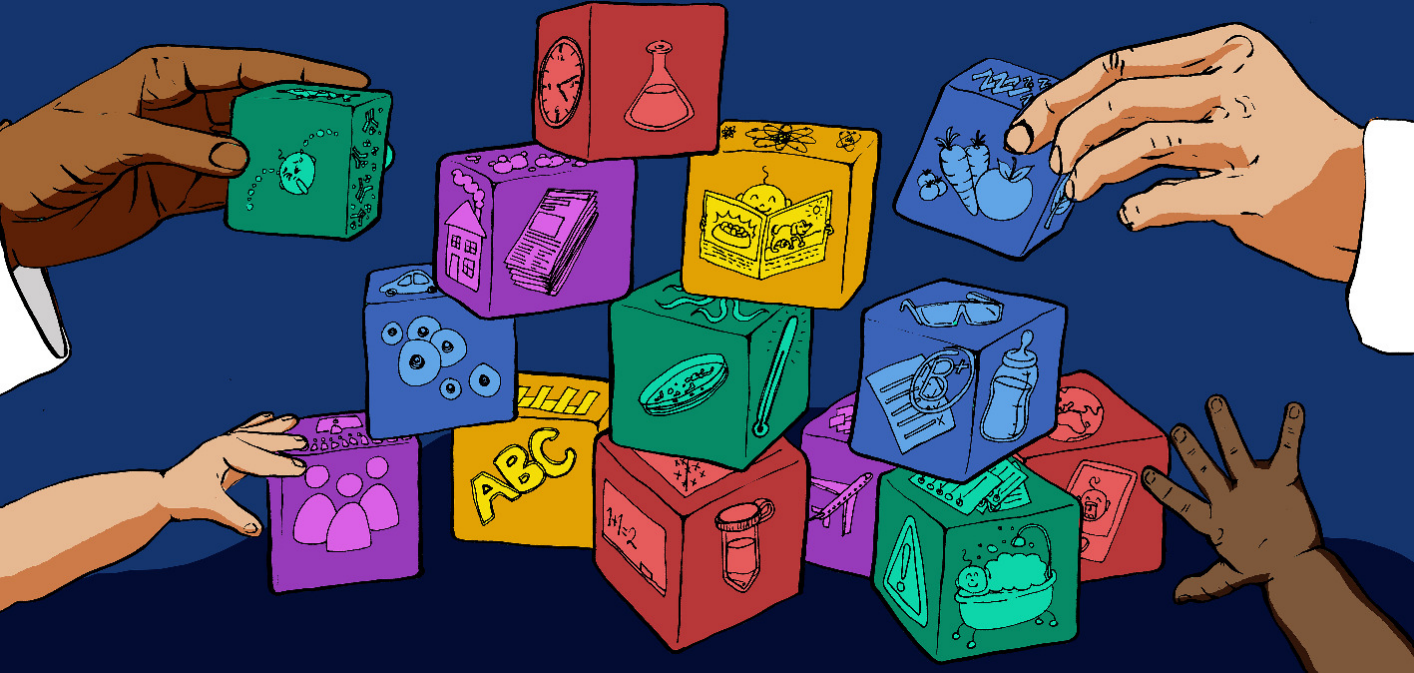
There are many sides to science and parenting, but they can be combined to build a successful career and happy family life.

IMAGE CREDIT: vividbiology.com.

## Travelling the distance

The perceived career benefits of mobility – particularly for early-career researchers – means that many scientists will bring up their children in a foreign country. In addition, short-term contracts can cause researchers to move their family repeatedly, leaving them far from their support networks.

In the course of this series we will feature several stories of researchers living or working in a different country to their children or partner for months at a time. Ali Twelvetrees, for example, had her child in Philadelphia in 2015, and moved to London in 2016 to complete her Fellowship; meanwhile her postdoc husband Dan remained in Philadelphia for another year to complete his project. “Emotionally and financially it was very hard”, says Ali. “I love being a mum, but not being able to share childcare with anyone in that period made the daily grind relentless”. Ali and Dan now have their own labs at the University of Sheffield.

Even when researchers can avoid emigrating, travelling for conferences and meetings still presents difficulties. Although some conference organizers are starting to offer funding and meeting rooms to help with childcare, many researchers are still faced with a difficult choice between spending time away from their children or losing out on networking opportunities. “I feel bad leaving my daughter alone with my husband for too long, but at the same time I feel bad rejecting invitations to conferences”, says Yanlan Mao of University College London. Trying to balance the two can produce extreme travel arrangements: Yanlan once travelled from the UK for a two-day trip to China.

## Flexibility is a double-edged sword

When asked what is the biggest advantage scientist parents have over parents in other careers, there was a clear consensus: flexibility. Most researchers are free to work when they like, although this benefit does come with a number of caveats. “My partner always says that academics have the freedom to work whichever 60 hours a week they choose”, says Adrian Liston of VIB in Belgium. In addition, certain tasks are better suited to flexible working than others. Grant applications and papers can be written at home, and it is often possible to perform computational research and data analysis remotely. However, many a planned wet lab experiment or field study has been postponed because of a sick child.

And while research can be performed at almost any time of day, children often have strict schedules: the start and end of the school or nursery day, for example, are inflexible. To accommodate leaving ‘early’ to pick up children, many parents resume work once the children are in bed. That said, several researchers mentioned to us that they struggle to leave work behind completely when spending time with their children. “Block those precious times with your kids off, and then when you actually do work, you will be more efficient” is Yanlan Mao’s advice. However, some parents told us that they cannot work at the same rate and intensity as they did before they had children.

## Better for everyone

Making scientific careers more family-friendly would benefit scientists without children too. The constant pressure to perform to get the next job and the next grant is experienced by all researchers, not just those who are also parents. By making it more acceptable to work flexibly – in the same institute for your whole career, if you choose – scientific careers will become more accessible to a diverse group of researchers, particularly those with other caring responsibilities and those with disabilities and chronic conditions.

A research career should not be a barrier to having children, and vice versa. “Research is not just a job”, says Gaby Da Silva Xavier. “I would not be me if I gave up on my work, and that arguably would not make me a good role model for my son”. As we hope to show in this series, having both is achievable and can enrich the lives of researchers and the people they work with.

## Note

This Feature Article is part of the Scientist and Parent collection.
